# A case report of pancreatic panniculitis due to acute pancreatitis with intraductal papillary mucinous neoplasm

**DOI:** 10.1186/s12876-020-01430-9

**Published:** 2020-08-24

**Authors:** Yuki Yamashita, Satoru Joshita, Tetsuya Ito, Masafumi Maruyama, Shuichi Wada, Takeji Umemura

**Affiliations:** 1 Department of Gastroenterology, Nagano Red Cross Hospital, Nagano, Japan; 2 Department of Medicine, Division of Gastroenterology and Hepatology, Shinshu University School of Medicine, 3-1-1 Asahi, Matsumoto, 390-8621 Japan

**Keywords:** Pancreatic panniculitis, Intraductal papillary mucinous neoplasm

## Abstract

**Background:**

Pancreatic panniculitis is a rare skin manifestation in pancreatic disease patients that most frequently develops on the lower legs. We report the unique case of a 68-year-old man who suffered from pancreatic panniculitis on his trunk associated with acute pancreatitis due to an intraductal papillary mucinous neoplasm.

**Case presentation:**

A 68-year-old man complained of a 2-day history of a tender subcutaneous nodule on his trunk. Laboratory tests and abdominal contrast computed tomography were consistent with acute pancreatitis due to an intraductal papillary mucinous neoplasm. A skin biopsy of the nodule histologically displayed lobular panniculitis with characteristic “ghost cells”, which indicated pancreatic panniculitis.

**Conclusions:**

In order to avoid a missed or delayed diagnosis, clinicians should bear in mind that pancreatic panniculitis can be the first manifestation of pancreatic disease when encountering subcutaneous nodules on the trunk.

## Background

Pancreatic panniculitis is a rare skin manifestation associated with pancreatic disorders that presents in approximately 3% of patients with acute or chronic pancreatic disease [1]. The disorder most commonly develops on the lower legs as ill-defined erythematous subcutaneous nodules. Such nodules are detected in up to 45% of patients with pancreatic panniculitis before recognition of the original pancreatic disease [1]. Accordingly, the chief complaint of the patient is sometimes erythematous nodule detection before abdominal symptoms. Clinicians therefore have the risk of overlooking the underlying pancreatic disease. We herein report the rare case of a 68-year-old man who had pancreatic panniculitis on his trunk associated with acute pancreatitis due to an intraductal papillary mucinous neoplasm (IPMN).

## Case presentation

**Fig. 1:**
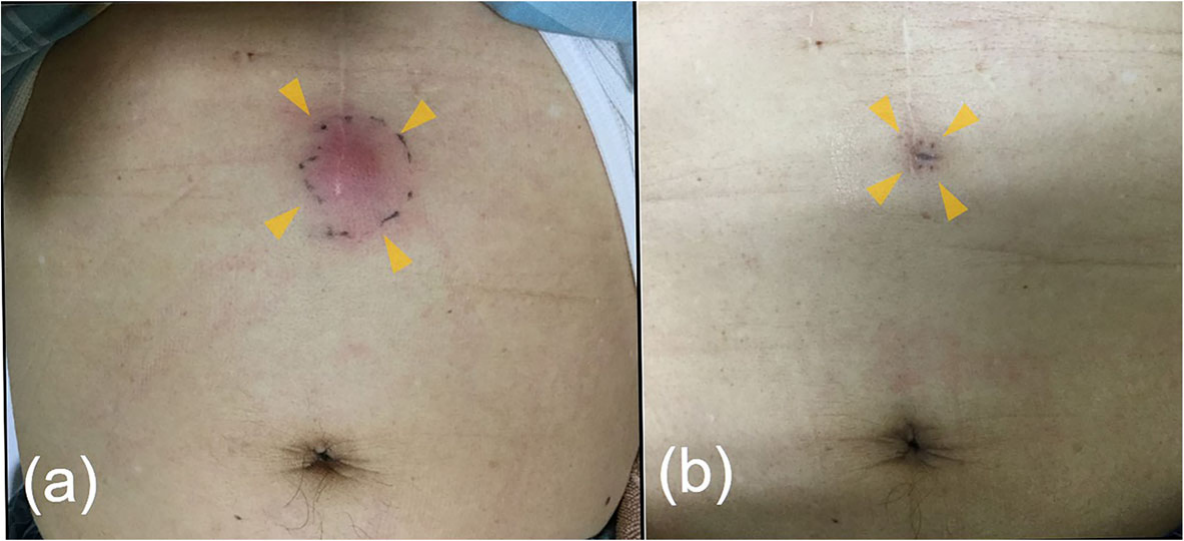
**a** An erythematous reddish subcutaneous nodule on the abdomen causing spontaneous pain and tenderness on admission (arrowheads). **b** The nodule disappeared 1 week after discharge, leaving only the biopsy scar (arrowheads)

**Fig. 2:**
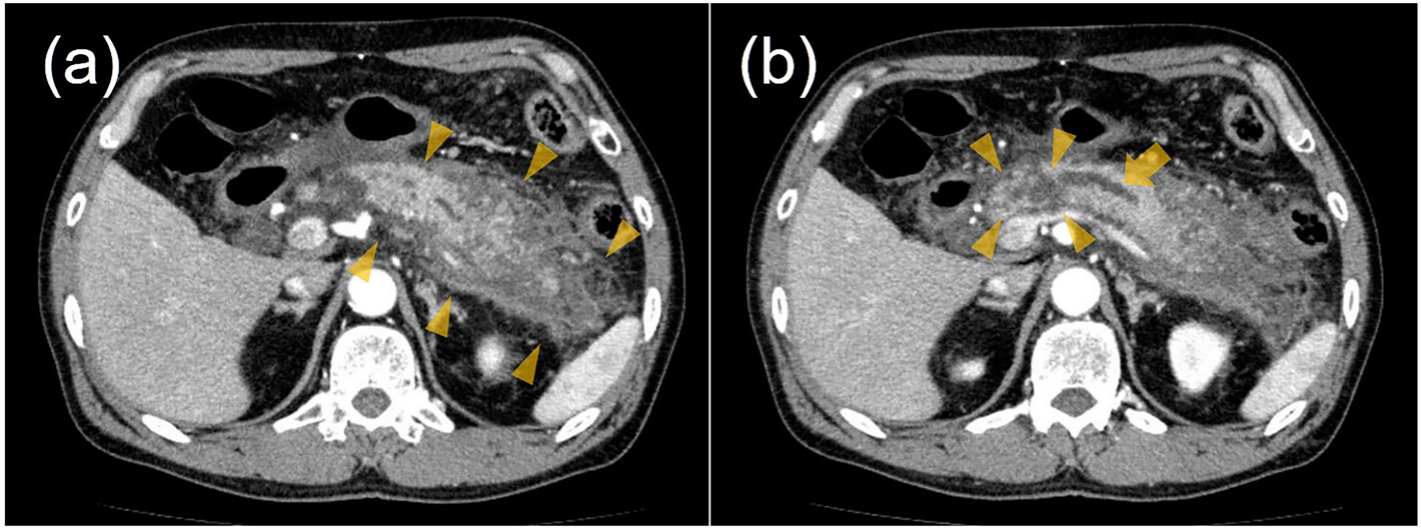
**a** Abdominal contrast CT on admission demonstrated a swollen pancreas with surrounding fat strands (arrowheads). **b** The CT showed a 20 mm multifocal cystic mass at the pancreatic body (arrowheads) along with an 8 mm dilation of the main pancreatic duct (arrow)

**Fig. 3:**
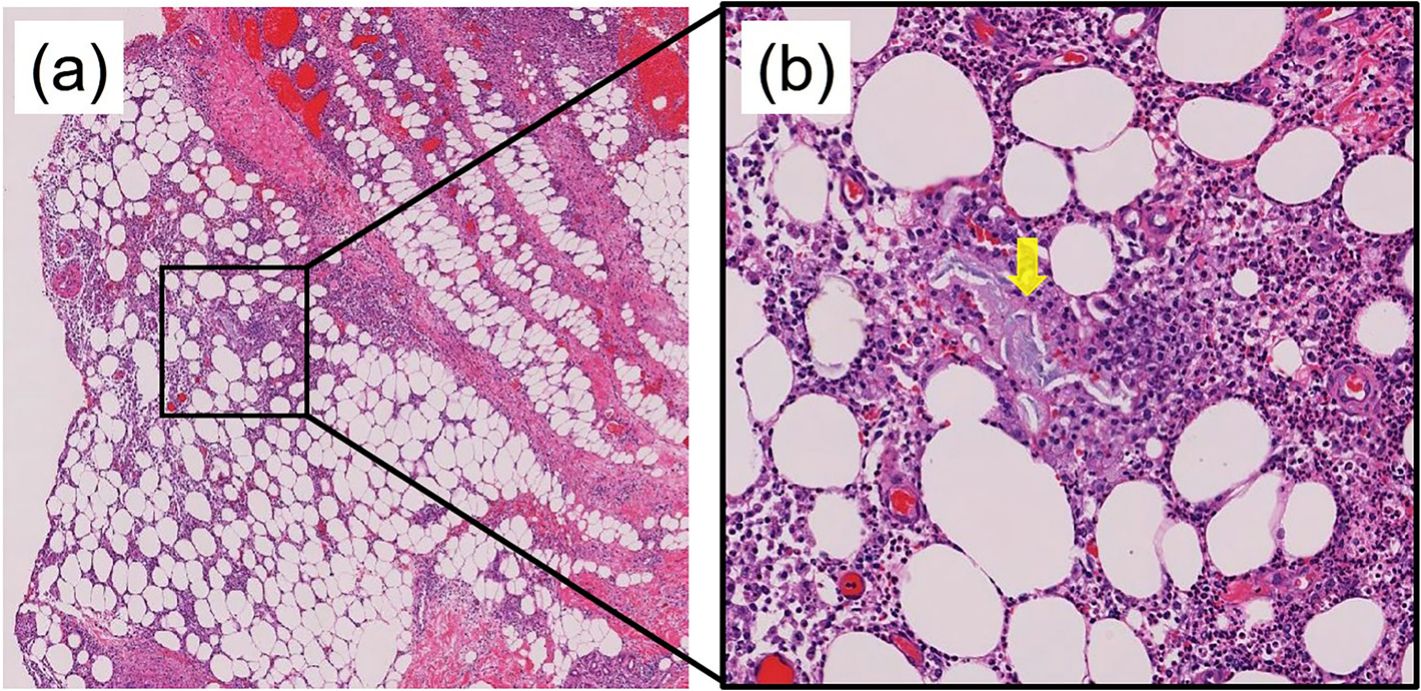
**a** A punched biopsy of the skin lesion revealed lobular panniculitis with focal necrosis of adipocytes. (hematoxylin and eosin staining, × 2 magnification. **b** The biopsy showed “ghost-like” cells with calcification surrounded by neutrophil-rich inflammatory infiltration (arrow). (hematoxylin and eosin staining, × 10 magnification)

A 68-year-old male patient was referred to our hospital by his primary care physician for further evaluation of a painful subcutaneous nodule on his upper middle abdomen, which was suspected to be abdominal cellulitis. Five days before admission, he had suffered from epigastralgia, nausea, and anorexia. Four days before admission, his epigastralgia had improved. Two days prior to admission, he noticed an erythematous nodule on his abdomen. His chief complaint on admission to our hospital was the painful nodule on his abdomen. He had been under medical treatment with aspirin, atorvastatin, colestimide, nicorandil, and famotidine for past medical histories of coronary artery bypass grafting (CABG), appendectomy, and dyslipidemia. He had no allergies. He had smoked 1 pack per day for 33 years before quitting 15 years earlier. He habitually drank 20 g of ethanol per day, with no history of heavy drinking. On examination, his temperature was 38.8 °C, blood pressure was 144/74 mmHg, and pulse was 98/min with regular rhythms. A painful and tender erythematous nodule was palpable on his epigastrium at the lower edge of a postoperative scar from CABG. The nodule was 2.5 cm in diameter and surrounded by pale erythema (Fig. 1a). Laboratory tests revealed a white blood cell count elevation of 15,650/μL along with a C-reactive protein (CRP) abnormality of 24.4 mg/dL, with no amylase or lipase elevation (Table 1). Abdominal contrast computed tomography (CT) confirmed grade 1 acute pancreatitis (Fig. 2a) and a 20 mm multifocal cystic mass at the pancreatic body along with an 8 mm dilation of the main pancreatic duct, which were compatible with a diagnosis of IPMN (Fig. 2b). Thereafter, he commenced intravenous fluid infusion and antibiotic therapy. On hospital day 3, he became afebrile and showed improvements in inflammatory clinical parameters. A punched biopsy of the skin lesion on hospital day 4 revealed lobular panniculitis without vasculitis findings. Histological analysis uncovered focal necrosis of adipocytes and “ghost-like” cells with calcification surrounded by neutrophil-rich inflammatory infiltration, which indicated pancreatic panniculitis (Fig. 3). Intravenous fluid infusion and antibiotic therapy were continued until discharge on hospital day 12 for nodule pain disappearance along with CRP normalization. The erythematous nodule on his abdomen disappeared 1 week afterwards (Fig. 1b). The patient has been complaint-free without nodule recurrence or additional nodule appearance since his discharge 2 years prior. His IPMN status is routinely monitored every 3 months by imaging studies.

**Table 1:** Laboratory data on admission

<Hematology>			<Chemistry>			<Tumor markers>		
While blood cell	15,650	/μL	Total Protein	6.0	g/dL	CEA	2.9	ng/mL
Neutrophil	84.5	%	Albumin	2.5	g/dL	CA19–9	2	U/mL
Red blood cell	504 × 10^4^	/μL	AST	27	U/L			
Hemoglobin	15.5	g/dL	ALT	31	U/L	<Arterial blood gas>		
Platelet count	20.7 × 10^4^	/μL	LDH	363	U/L	pH	7.503	
			ALP	357	U/L	pCO_2_	29.3	mmHg
<Coagulation>			GGT	75	U/L	pO_2_	62.1	mmHg
Prothrombin %	90	%	Total Bilirubin	1.0	mg/dL	HCO_3_^−^	22.8	mmol/L
PT-INR	1.06		BUN	15.3	mg/dL	BE	1.1	mmol/L
Activated partial thromboplastin time	31.8	sec	Cre	0.73	mg/dL	Lactate	0.6	mmol/L
			Na	135	mEq/L			
			K	3.3	mEq/L			
			cCa	9.2	mEq/L			
			AMY	73	U/L			
			Lipase	38	U/L			
			CRP	24.4	mg/dL			

Abbreviations: *Alb* Albumin; *ALP* Alkaline phosphatase; *ALT* Alanine aminotransferase; *AMY* Amylase; *APTT* Activated partial thromboplastin time; *AST* Aspartate aminotransferase; *BE* Base excess; *BUN* Blood urea nitrogen; *CA19–9* Carbohydrate antigen 19–9; *cCa* Corrected calcium; *CEA* Carcinoembryonic antigen; *Cre* Creatinine; *CRP* C-reactive protein; *GGTP* Gamma-glutamyltranspeptidase; *Hb* Hemoglobin; *LDH* Lactate dehydrogenase; *Neut*, neutrophils; *PT*, prothrombin time; *INR* International normalized ratio; *T-Bil* Total bilirubin; *TP* Total protein; *RBC* Red blood cells; *WBC* White blood cells

## Discussion and conclusions

Pancreatic panniculitis is a rare skin manifestation associated with pancreatic disease. In the clinical setting, panniculitis is noticed as erythematous, ill-defined, and/or red-brown nodules [2] that generally affect the lower limbs and buttocks, rarely the trunk and upper extremities [3]. Only 9 cases have been described of pancreatic panniculitis on the trunk [3–11]. We encountered a unique case of subcutaneous nodules on the abdomen with latent IPMN. Skin biopsy revealed the typical histological findings of pancreatic panniculitis of lobular neutrophilic necrotizing panniculitis intermingled with specific necrotic anucleate adipocytes, called “ghost cells” [12].

The mechanism of pancreatic panniculitis onset remains unknown. It is hypothesized that systemically released pancreatic enzymes such as amylase and lipase can cause lipolysis and fat necrosis [13, 14], resulting in pancreatic panniculitis as a specific clinical phenotype. However, several cases of pancreatic panniculitis with normal serum pancreatic enzymes have been reported [15, 16], as in the present case. One possible reason for our patient was that his pancreatic amylase had already peaked due to pancreatitis improvement since such pancreatitis symptoms as epigastralgia had improved 4 days before admission. Therefore, it is clinically important to consider pancreatic panniculitis in patients with subcutaneous nodules even in the absence of abdominal symptoms for underlying pancreatic disorders, regardless of pancreatic enzyme status. This may avoid a missed or significantly delayed diagnosis of primary pancreatic disease.

The main pancreatic diseases related to pancreatic panniculitis have been reported as acute or chronic pancreatitis, pancreatic carcinoma (ductal adenocarcinoma, acinar cell carcinoma, or neuroendocrine carcinoma), and IPMN. In the present case, the underlying pancreatic disease was acute pancreatitis due to IPMN. Zundler et al. reviewed that subcutaneous lesions were noted prior to the diagnosis of pancreatic disease in 48.9% of reported cases [3]. In terms of clinical course, the period between subcutaneous lesion appearance and the detection of abdominal disorders could be several months for pancreatic panniculitis [17]. Table 2 summarizes the literature on pancreatic panniculitis displaying subcutaneous nodules as a chief complaint before diagnosing pancreatic disease. We searched the English-written literature between 1994 and 2019 using the parameters “pancreatic panniculitis” and “subcutaneous fat necrosis AND pancreas” in PubMed to identify 56 reported cases. The site of the nodules was predominantly the leg, with some cases on the trunk or arm. It should be noted that the period between subcutaneous lesion appearance and detection of the pancreatic disease was up to 48 weeks, and the underlying pancreatic disorder was a pancreatic neoplasm in nearly half of patients. Of all reported cases summarized in Table 2, only 2 were described as latent IPMN presenting as pancreatic panniculitis [22, 41]. Pancreatic panniculitis could therefore serve as the impetus for an intensive search for pancreatic neoplasms in order to prevent a potentially long delay in diagnosis [17, 60].

**Table 2:** Reported cases of pancreatic panniculitis with subcutaneous nodule(s) as a chief complaint

Age (years)	Sex	Nodule site	Period preceding diagnosis (weeks)	Amylase (U/L)	Lipase (U/L)	Pancreatic disease	Reference
77	M	Leg	2	WNL	6027	Pancreatic carcinoma	[18]
64	M	Leg	3	3052	6205	Pancreatic acinar cell carcinoma	[19]
50	M	Leg	4	111	327,500	Pancreatic acinar cell carcinoma	[20]
67	M	Trunk, leg	0	4544	3885	Chronic pancreatitis	[4]
57	M	Leg	16	2161	27,575	Pancreatic carcinoma	[21]
61	F	Leg	12	148	N/D	IPMN	[22]
76	M	Leg	1	2000	400	Acute pancreatitis	[23]
34	F	Leg	2	650	2893	Pancreas allograft rejection	[24]
73	F	Trunk, leg	8	WNL	14,747	Pancreatic acinar cell carcinoma	[3]
86	F	Leg	4	N/D	870	Pancreatic carcinoma	[25]
55	M	Leg	4	48	N/D	Pancreatic neuroendocrine carcinoma	[26]
63	M	Arm, leg	0.3	6647	3000	Acute pancreatitis	[12]
49	M	Leg	4	Over NL	N/D	Acute pancreatitis	[27]
71	F	Leg	4	1073	1871	Chronic pancreatitis	[28]
66	F	Leg	4	N/D	3000	Acute pancreatitis	[29]
54	M	Leg	36	WNL	9018	Pancreatic acinar cell carcinoma	[30]
2	M	Trunk, leg	0.7	430	N/D	Acute pancreatitis	[5]
35	M	Leg, buttock	4	3 × ULN	N/D	Chronic pancreatitis	[31]
50	M	Leg	3	1909	2306	Acute pancreatitis	[17]
79	F	Leg	16	WNL	N/D	Pancreatic acinar cell carcinoma	[32]
34	M	Trunk, leg	4	4900	1400	Chronic pancreatitis	[6]
49	M	Trunk, arm	0	1248	N/D	Acute pancreatitis	[7]
61	F	Arm, leg	0	241	515	Acute pancreatitis	[33]
39	M	Trunk, leg	2	Over NL	Over NL	Chronic pancreatitis	[8]
17	F	Leg	0.3	829	1330	Acute pancreatitis	[34]
63	F	Leg	16	WNL	8000	Metastatic pancreatic adenocarcinoma	[35]
82	M	Leg	2	Over NL	Over NL	Pancreatic acinar cell carcinoma	[36]
18	F	Leg	0	163	1333	Acute pancreatitis	[37]
69	M	Leg	8	9	41,405	Metastatic pancreatic adenocarcinoma	[38]
79	M	Leg	8	WNL	4668	Metastatic pancreatic NEC	[39]
68	M	Leg	0	41	16,022	Metastatic pancreatic NEC	[40]
75	M	Leg	0	54	24,060	Pancreatic adenocarcinoma	[40]
67	F	Leg	10	N/D	N/D	IPMN	[41]
72	M	Leg	12	N/D	Over NL	Pancreatic NEC	[42]
42	F	Leg	2.8	1983	3550	Acute pancreatitis	[43]
53	M	Leg	0	2252	16,000	Acute pancreatitis	[44]
59	F	Leg	0	3000	N/D	Acute pancreatitis	[45]
81	M	Leg, trunk, buttock	48	WNL	6430	Pancreatic acinar cell carcinoma	[9]
49	M	Leg	4	Over NL	N/D	Acute pancreatitis	[27]
66	M	Leg	2	2844	6265	Pancreatic acinar cell carcinoma	[46]
69	M	Leg	6	WNL	1326	Pancreatic acinar cell carcinoma	[47]
75	M	Leg	20	WNL	62,650	Pancreatic tumor	[48]
39	M	Leg	1	363	1355	Chronic pancreatitis with pseudocyst	[48]
54	F	Leg	16	1815	11,935	Chronic pancreatitis	[49]
47	F	Leg	2	N/D	562	Acute pancreatitis	[50]
46	F	Leg	0	Over NL	N/D	Chronic pancreatitis	[51]
61	F	Arm, leg	0	WNL	13,510	Metastatic acinar cell carcinoma	[52]
77	F	Leg	1	4810	4901	Acute pancreatitis	[53]
68	M	Leg	32	4700	16,000	Chronic pancreatitis	[54]
19	F	Trunk, leg	0	869	759	Solid pseudopapillary tumor of the pancreas	[10]
54	M	Arm, leg	0.6	1036	7231	Acute pancreatitis	[55]
76	M	Trunk	0	2461	2488	Acute pancreatitis	[11]
82	M	Arm, leg	48	45	7674	Pancreatic tumor	[56]
47	M	Leg	2	1539	1516	Pancreatic adenocarcinoma	[57]
66	M	Leg	1	2036	3538	Acute pancreatitis	[58]
48	M	Leg	1	4500	3500	Chronic pancreatitis	[59]
68	M	Trunk	0.3	73	N/D	IPMN	Present case

Abbreviations: *M* Male; *F* Female; *N/D* Not described; *Over NL* Over normal limit; *ULN* Upper limit of normal; *IPMN* Intra-ductal papillary mucinous neoplasm; *WNL* Within normal limit; *NEC* Neuroendocrine carcinoma

In conclusion, clinicians should bear in mind that pancreatic panniculitis can be the chief complaint of pancreatic disease when encountering subcutaneous nodules on the trunk to prevent a missed or delayed diagnosis.

## Data Availability

N/A

## Abbreviations

Alb: Albumin
ALP: Alkaline phosphatase
ALT: Alanine aminotransferase
AMY: Amylase
APTT: Activated partial thromboplastin time
AST: Aspartate aminotransferase
BE: Base excess
BUN: Blood urea nitrogen
CABG: Coronary artery bypass grafting
CA19–9: Carbohydrate antigen 19–9
cCa: Corrected calcium
CEA: Carcinoembryonic antigen
Cre: Creatinine
CRP: C-reactive protein
CT: Computed tomography
GGTP: Gamma-glutamyltranspeptidase
Hb: Hemoglobin
INR: International normalized ratio
IPMN: Intraductal papillary mucinous neoplasm
LDH: Lactate dehydrogenase
Neut: Neutrophils
N/D: No data
Over NL: Over normal limit
PT: Prothrombin time
RBC: Red blood cells
T-Bil: Total bilirubin
TP: Total protein
ULN: Upper limit of normal
WBC: White blood cells
WNL: Within normal limit
